# The Doctor Analyzed

**Published:** 1904-08-15

**Authors:** J. W. Clemmer

**Affiliations:** Columbus, Ohio


### THE DOCTOR ANALYZED.

BY J. W. CLEMMER, M.D., COLUMBUS, OHIO.

Read before the Ohio State Dental Society, December, 1903, and printed from advance proofs by courtesy of "Dental Summary."

Medicine is a profession of specialties. Dentistry was the first to establish itself. It has long since assumed the proportions and worth of a distinctive profession. As an art it must remain distinctive; as a science it belongs to medicine. This relationship is emphasized for the purpose of this paper, under whose caption the writer has the temerity to attempt an analysis of that composite medical scientist known as the doctor.

The subject will be discussed under the following classifications:

1. The doctor's entrance upon professional work.
2. The relation of the doctor to the professional.
3. The doctor as related to society.
4. The doctor's duty to himself.
5. The doctor as a diagnostician.
6. The good and the bad in the doctor as a result of professional work.
7. Nature as a teacher and the ideal doctor.

### HOW THE DOCTOR ENTERS UPON PROFESSIONAL WORK.

The proudest, happiest moment in the doctor's life is when, on commencement day, diploma in hand, he receives the blessings and encomiums of his teachers. He receives the hand of fellowship and is welcomed to the profession. He is told that he is about to enter a calling full of grand possibilities, dignities and honor. Achievement to the neophite appears as easy to gather from future propects as fall Pippins from the orchard at the old home.

After these memorable events, the doctor undertakes the solution of the momentous questions of a location and an office sign to guide a waiting public. As time goes on he finds that the particular location of the office does not make so much difference as what he puts in the office. It requires time and the sacrifice of pedantry, however, to learn that the office requires something more than fine furniture and a display of instruments and trappings. He finds that the laboratory and operating-room should be furnished with the best-directed means and methods to relieve human suffering. They require tact and talent, patience and skill. This furniture comes from the great store-house of wisdom, developed in college curriculum and polished in the school of experience. The question as to whether the doctor shall wear a white duck office-coat is not so important as whether the coat shall be maintained in its native color, even at the risk at an extra laundry bill. If "cleanliness is next to godliness," in a dental office, dirt is devilish and soap a virtue. The ideal surgeon is characterized by an intimate acquaintance with the nail brush. The advance in scientific knowledge among the laity will not tolerate faulty technic in professional hands. The completeness with which asepsis is carried into effect is a sharp index to the character of the operator and his work.

### THE RELATION OF THE DOCTOR TO THE PROFESSION.

There are doctors and doctors, all kinds of men engaged in professional work. Some stand in relation to their work as artists and scientists, developing new facts and methods, thus elevating the profession. They have the dignity and worth of professional gentlemen. They engage in their work, not as a trade, but as a profession worthy of their best effort. There are others who regard the profession simply as a means of livelihood. They plod along and dress like ordinary craftsmen. They look upon their work as so much commerce, and resort to the ways of shopmen in using the arts of advertisement. Doctors with commercial standards in professional work may not see any impropriety in advertising, but in so doing they over-reach bounds of individual refinement, not to mention the breach of professional ethics, when it is remembered that in advertising they are not calling attention to ordinary commodities, as stoves or hats, but to their personal qualifications. There is a difference as to whether personal tact and talent or merchandise is proclaimed in public print. The doctor may announce his profession, location or removal without violating the rules of good conduct, but when he advertises his accomplishments he ditches his profession, and unhorses himself. There is still another advertiser of the "blow-hard" type, not less reprehensible than the first. He exploits in the public press, under guise of news without paying the usual rates, his attendance upon noted people, his operations, his methods, instruments, discoveries, etc. He discusses on the street corners his patients, their diseases, and his skill in their relief. No opportunity is lost to exercise his brazen egotism.

The organization work of the profession requires united effort to keep the colleges, the societies, journals and its general literature up to the highest standard.

Membership in any organization assumes unavoidable responsibility. The doctor accepts, with his license to practice, this responsibility which he cannot avoid.

No man is free to act under moral or civil law, unless he chooses to do right. He can employ natural forces only as he works in accord with natural law.

Thus it is with the doctor and the profession. He cannot enter it without accepting the conditions imposed. He could not join a company of bandits and do less. Organization means compliance with rules of conduct.

The profession has both written and unwritten laws to govern the conduct of its members. The written laws are intended only for the "breachy" doctor. The unwritten laws are innate with every true gentleman who needs no sign-board to direct him in right relations to his fellows and the public. A prerequisite of a member of the profession is that he be a gentleman; nothing more, nothing less, can be exacted in a code of ethics.

### THE DOCTOR AS RELATED TO SOCIETY.

The profession owes society a consideration in every community. The doctor should advise against evils which the greed, ignorance or carelessness of individuals inflict upon the public. His training naturally qualifies him to serve the public as a sanitarian. Every doctor should be interested in the work of municipal sanitation. There are many ways in which he can serve the needy and afflicted by assisting the work of charity. He can advance the work of education by serving on the school board. Should the doctor accept a political position, in local affairs, let it be fortified by a strong manhood, honor and honesty. His name should be a synonym for good government. The doctor should take interest in public affairs as a representative citizen, but he should avoid partizan politics.

The doctor should make the social defence against the various systems of faith-healers by accepting the bit o truth found in each, when the balance will sink out of sight by the weight of its own folly.

### THE DOCTOR'S DUTY TO HIMSELF.

Doctors, as a rule, neglect the business side of their work. They give their energies to the scientific and philanthropic aspects of their calling, very much to the neglect of their bank accounts. The public understands this, and the unscrupulous ply the arts of the imposter. The doctor is a poor collector and often a poor man. He dislikes the financial relation between himself and his patient; hence he accumulates a long list of unpaid accounts which at the end of his career constitute a large part of his estate. The experience of past generations of doctors would teach those who follow the words of wisdom. Gratitude, compensation and patronage are often lost to the lack of business methods. Is it not true, gentlemen, that a bill is often lost because its payment is not urged at the proper time? Is it not true that patients cease your patronage because they owe an old bill? Is it not true that the patient who has quit you, because he owes you, has lost gratitude for the work already done and even dislikes you? One of two things should happen. Let me advise: Either collect your bills or hang the ungrateful debtor. Be governed by business methods rather than by sentiment. You must recognize the value of your services if you would have your patients appreciate and pay for them.

### THE DOCTOR AS A DIAGNOSTICIAN.

The doctor's reputation often hinges upon his ability as a diagnostician. Diagnosis depends largely upon accuracy of observation and thorough examination. The patient will not deprecate the doctor's ability if he makes repeated examinations before offering an opinion; but if the diagnosis proves faulty, his reputation will suffer. An important factor in making a diagnosis and, incidentally, an element in the acquisition of an extensive practice, is the tact and gentleness with which the doctor examines his patient. The first visit of the patient is likely to be full of fear and apprehension. Such a patient requires more than anything else to be assured that his fears are unfounded. Tact and patience on the part of the examiner are necessary to inspire confidence. This bit of diplomacy is necessary to a thorough examination; without it the diagnosis and the patient may both go astray. Only a few minutes are required to win the confidence and patronage of the patient, by inquiry into his subjective condition and the events that induced him to seek relief. The most successful practitioner is he who inspires the most confidence and conducts his examinations with the greatest gentleness and tact consistent with thoroughness. The most unpromising of neurotic patients will become easily managed, if only the practitioner will consider the psychological side of his subject. The office of the practitioner is the play-ground of mental influence. How often has toothache ceased before the patient mounted the operating chair? Suggestions of relief were enforced by every event in relation to the patient's visit. The doctor is constantly offering suggestions that are accepted by the patient. Positive, repeated suggestions will accomplish much. Try it.

### THE GOOD AND THE BAD IN THE DOCTOR AS A RESULT OF PROFESSIONAL WORK.

There is something in the nature of professional work to change and modify the native characteristics of its devotees. The environment and association of the doctor have an exaggerated influence upon his social and moral life. This influence works upon a sliding scale between good and evil. The doctor is both good and bad. There is no intended division of practitioners into two classes with all the good in one and all the bad in the other. All human nature partakes of these opposite qualities.

The life of the practitioner, from the nature of his work, develops both sunshine and storm. The doctor represents the best and worst of men. At times the magnanimity and philantropy of his profession takes possession of his heart and he is willing to make any sacrifice for the welfare of his patient. At other times he would rather see the patient die under his treatment than get well in the hands of another physician. He would rather see his patient suffer an aching tooth under his treatment than get relief at the hands of his neighbor dentist. A close analysis of the doctor sees him tender and severe, forgiving and resentful, charitable and selfish, generous and unmerciful, liberal and envious, fair-minded and prejudiced. Doctors are known to be gratified to see their failures turned to success by another. Others would rather read the obituary of a patient than to see him carry his failure to another practitioner.

The doctor's love may be the purest or his hate the deadliest, his friendship the strongest or his enmity the most severe. What is it in the doctor's calling that develops such opposite qualities? What can be eliminated from his education or work that makes him so devilish? His professional work is more artful than ever before. There must be some means to produce a corresponding improvement in his Jekyll-Hyde disposition. His judgment along scientific lines has a high degree of perfection in the healing art, but his sensibilities have had no training, resulting in a one-sided development.

The trouble grows out of the fact that professional education contemplates only physical phenomena. We should consider the spiritual side of man. Psychology and moral philosophy must enter the problem, if we would extricate ourselves from this embarrassing situation.

The fundamental laws of nature are as simple as they are universal in their application to details. One of these is the law of vibration or attraction, of which gravity is is only a part. In this way all manner of like associations are formed. Minerals are massed, birds flock, animals herd, men associate for good or evil. Like attracts like. Love, hope, fear, hate malice, envy—all multiply, each after its kind. Smiles and tears are contagious.

The law applied in the problem in hand, it is found that when man is physically disordered by disease, the spiritual man is morbid. The spiritual functions, having to mediate through the physical organism, manifest a corresponding perversion. It is the life-work of the doctor to bring harmony out of disorder, to comprehend the pathological state and cure morbid processes. The patient is unhappy and wears the gloomy garments of discontent. In this depressing atmosphere the doctor, day after day, is subjected to a contagious influence of mental or moral aberration—a fact not usually appreciated.

By education and experience the doctor is able to guard himself against contagion of physical disease. He is prepared to avoid inoculation or infection. He understands sanitation and disinfection and is thus exempt from physical danger.

Unfortunately, his education is incomplete. The colleges failed to teach him the physiology and pathology of the spiritual man as they did of the physical man. They taught him to avoid communicable diseases of the body, but gave him no instructions as to the contagion of moral sensibilities. He was not instructed against the contaminations of mental and moral aberrations, as bigotry and intolerance, deceit and treachery. The student learns bacteriology, antisepsis and disinfection, but is untutored as to the microbes of scandal and misanthropy. He is taught that physical disorder is unnatural. He should understand that it is just as evil to violate spiritual law as it is to violate physical law; that moral and social distempers are just as unnatural as physical disorder; that it is no more natural to lie than it is to limp, to steal than it is to cough, to hate than it is to have decayed teeth.

The spiritual man being so neglected, it is not strange that doctors should manifest the various forms of selfishness, that they are envious and resentful, that they do not get on together as partners, that they are jealous. The wonder is that they are so kind, so good, so charitable.

The great desideratum in the doctor's life is the supreme art to which all others pay tribute—the art of living. To know how to live happily and wisely is the highest accomplishment in human experience.

### NATURE AS A TEACHER AND THE IDEAL DOCTOR.

Greatness is characterized not so much by acquirement as by development. Many men find knowledge but do not discover themselves. Man is a unit in the order of nature. He is united with a series of natural phenomena. In order to comprehend his relation to the world about him, he must know something of his own existence, as related to the vital energies of nature. He must accept nature as a teacher to unfold his own life and amplify the meaning of individual experience.

This is culture, pure and simple; knowledge makes one acquainted with facts, nothing more; it is circumspective, scientific. Culture is introspective and philosophic. Knowledge is academic and mediates through the acquirement of facts. Wisdom comes from the great school of nature and is evolved out of experience.

There is a great principle behind nature working through immutable laws and forces, century after century, as witnessed in the phenomena and processes of its vital energies. The one great problem of man is to unfold and comprehend this principle and establish his relation to it. This grand thought or order of nature is being gradually revealed to the observation of man.

When men study the works of nature as they study the works of art, they find hidden back of the natural forms, as flower, tree, river, cloud and star, the vital principle or Great Artist, working through immutable forces and laws, in accord with which are evolved the wonderful productions of growth and development in the matchless splendor of color, design, harmony and beauty.

As God is the Supreme Artist, so the highest art in man is the art of living—living in recognition of his place in the order of nature and his relation to the Great Artist. The wonderful works of nature from microbe to the sun, with their kaleidoscopic beauty, harmony and power, constitute the laboratory of nature and reveal how God works in the universe.

The dental laboratory turns out a work of art that is a close duplicate of nature's form, color and use. The dentist demonstrates that thoughts are forces and have creative power. Artificial teeth have their origin in the thought-forces of the dentist before they receive their natural expression or embodiment. Before the creation of natural teeth they were conceived in the thought-forces of the great creative principle. Art copies after the great Artist. The dentist in making a set of teeth imitates nature. His ideals, his models and his ambition conform to natural processes.

Man's noblest impulses and highest inspirations, whether his energies take to music, mechanics, fine art, professional or industrial life, have their origin in the perfect work of nature. There are no ideals or inspirations above nature, either for physical or spiritual development. Good workmanship must have an ideal. Idealism is man's highest conception of truth.

The unattainable and the unknowable often reduce his ideals to a symbolic representation. His attainments are beset by limitations. As the limits of knowledge are extended the ideals become higher and we approach nearer to the heart of nature. As nature is made to yield her secrets, advancing generations of men reduce mystery and symbolism into conformity with truth.

This may be said in all pursuits in life. When the dentist masters the problem of heredity and the things we call dyscrasia, diathesis, morbid influences, both local and systemic, he will find he is getting acquainted, not so much with his patient, as with nature. As the doctor looks to nature for his ideals and models, as he imitates her in the most exact methods, and as he is proud of his accomplishments of the physical plane, so the doctor may look, likewise, to nature for his ideals and accomplishments in the development and betterment of his spiritual life. When the doctor follows the teaching of nature on the spiritual plane as he does on the physical, his social and moral obliquities will be replaced by the virtue and reward of correct living. Envy, hatred, malice and all forms of selfishness will dissolve out of his work and his life by the softening influences of altruism.

Such thoughts furnish the doctor with a stimulus to wise deliberation. They add wisdom to professional erudition. They lead to the highest form of education, because they aid self-discovery and interpret the meaning of experience in its proper relation to others. Such thoughts correct selfish propensities and go to establish the brotherhood of man. This teaching of nature affords a correct philosophy, because it puts man in command of the order of the universe. It is the basis of true religion, because it establishes a close relationship with the Divine Spirit.

### DISCUSSION.

DR. J. S. CASSIDY: The chairman of your executive committee notified me some time ago that I was down for discussion of a paper the subject of which was "The Doctor Analyzed." I now realize that the discussion is a little hard after hearing the excellent paper of Doctor Clemmer, and the principal reason for the hardness of the discussion is owing to the fact that I cannot find anything in the Doctor's paper I can oppose very strenuously. However, in order to keep in touch with the old popular idea that doctors cannot agree thoroughly on any subject, I will say that I think his paper is more on the synthetical order than on the analytical. The essayist does not take up his task by dissecting his subject, he rather builds it up piece by piece, and all elements are so well chosen, that the complete subject, the ideal doctor, was made a very perfect man, indeed. This ideal doctor, as presented by the accomplished architect, is most considerate toward his professional brethren; he is hopeful, always kind and considerate to them; he is always solicitous for the welfare of his patients; he confines himself to the printed code of ethics that we have talked about. He takes as his motto the Golden Rule that Doctor Wright was trying to quote this morning—"Do unto others as you would have them do unto you," and do it first.

The essayist also said that doctors should be just to themselves; that is, they should require prompt and reasonable recompense for their services. Now, that is a point where a great deal of prudence should be exercised. I have known bills to run along for ten or fifteen years and they were finally paid in full, whereas, if they had been urged too strenuously, those bills would not have been paid at all. He should be prudent in that matter. There might be a system adopted such as shown to us this morning by Doctor Smith. It is a good idea for the doctor to be just to himself.

There is another thing in which I cannot fully agree with the essayist. I am sure that the practice of medicine *per se* does not influence the doctor to devilish acts at times. My impression is that the practice of medicine in any of its branches is more uplifting and more helpful to poor humanity than any other calling, save one, and a majority of us here who are dentists, I presume, will agree with me in this statement. Is it true that a Mr. Hyde exists more or less in every doctor? Does he exist in the doctor more than other people? I think not.

The essayist suggests that to protect this devilish nature which sometimes gets in doctors, that the study of psychology may help him out in that respect. That is very true, and it is very much to his advantage to have studied psychology, and to keep it up; but unfortunately with the doctor more than with other persons, he is compelled to study and understand more or less of the physical science and studies of psychology and moral philosophy from the standpoint of the materialist; thus entirely, but unconsciously, perhaps, ignoring the existence of the human soul.

I want to thank the Doctor for his paper, and I want to thank him especially for his allusion to the importance of the study of the laws of vibration. There is no natural phenomenon that is not caused by or consequent to vibration. It is vibration by which we can conceive of the sweetness and beauty of the rose, the harmony of music and the grandeur of electricity and heat and light.

Dr. W. H. Whitslar, Cleveland: Herodotus, one of the earliest writers among medical men, mentions the various specialties of medicine practiced during his time. He mentions specialists for the care of the stomach, the eyes and the teeth, thus early recognizing dentistry as a specialty of medicine.

For centuries the growth of this specialty seems to have been stunted, as we hear nothing during this period that tends to show any progress of the aforesaid art.

Climatic conditions, the intermixture of races and the diversity of foods have made the care of the teeth a necessity and it remained for the enterprising and progressive Americans to develop the principles of dentistry to circumvent the ravages of decay.

We all know of the difficulties that Dr. Chapin A. Harris encountered in his endeavor to start a dental college. Fifty-two years after his struggles the American Medical Association at Richmond, Va., organized a section in stomatology as a specialty *per se* of medicine. This met with the hearty approval of such men as Dr. Samuel D. Gross, of Philadelphia, Sayre, of New York, and Dr. Nathan S. Davis, of Chicago. This section at the present time is highly honored by the profession.

While dentistry may be recognized as a specialty of medicine, one fact is paramount—it is the only specialty of medicine that grants a degree without a knowledge of medicine in the general.

A. D. D. S. permits a practitioner to practice dentistry only, while any other specialist of medicine may at any time, if he so desires, become a general practitioner. This fact is made more forcible in foreign countries than in the United States, some countries refusing the title of doctor to one whose degree includes only the D. D. S.

Doctor Clemmer, in his paper, makes a very clever distinction when he says, "As an art, it must remain distinctive; as a science it belongs to medicine." The old-time jest at the expense of the profession that "A doctor may bury his blunders," surely does not apply to the dentist. He finds them staring him in the face when he would be glad to disown them and crying out at him from dying nerves and turbulent abscesses.

Dentistry, as an art, has made most wonderful progress within the last quarter of a century. An art is greatest when it most nearly simulates nature, and with the advent of porcelain on the scene, there seems but little required to complete the claims of dentistry as an aspirant for a position of honor among the arts.

Doctor Clemmer has given us a comprehensive view of the dentist's life and environment, developed, as he says, in the college curriculum and polished in the school of experience.

How true it is that education does not consist of rules and principles learned in our schools and colleges, but rather of the application of those rules and principles to our daily needs.

In my capacity as teacher it has been my experience on various occasions to receive letters from graduates who, on beginning to make practical use of the principles taught in the school, remark that it is a very different thing to stand under a demonstrator and perform an operation under his instructions, than it is to tackle it all alone without advice from an experienced source.

This responsibility is the first chapter in his dental education.

Occasionally we hear a newly-fledged dentist say he has learned more by experience than was ever taught him at college, little realizing that the experience mentioned is but the application of the knowledge he gained while at school.

The association of progressive dentists, each seeking all the good he can find for the advancement of his profession, cannot but be elevating and ennobling, and tends to eliminate from the profession the petty *jealousies* so frequently found among members of the same calling.

A code of ethics is not a necessity if we live above its requirements, but it serves as a check to those individuals whose nature might tempt them a little beyond the margins of ethical confines.

A dental society enlarges, rather than restricts, one's opportunities, as any interchange of ideas must benefit the individuals of a community. A dentist's close contact with individuals in his professional capacity tends to develop in him the proper delineation of character and he becomes unusually observant.

Let us all get ourselves under complete control, for, by calmness, tact, patience and confidence in our ability to master the case at hand, we inspire confidence in our patients and the battle is half won.

The spiritual aspect of the Doctor's paper has especially appealed to me, for the desire to alleviate suffering and minister to the afflictions of our fellow-beings brings us closer to the Divine Being whose mission on earth was to alleviate the woes of mankind. Personally, I wish to thank Doctor Clemmer for this very able paper which is a heart-to-heart talk to the profession, and I am sure it has been greatly enjoyed by all present.

